# Describing the patient trajectory for unplanned care in a region of Flanders, Belgium: A prospective observational study

**DOI:** 10.1080/13814788.2026.2745925

**Published:** 2026-10-26

**Authors:** Patrick Van de Voorde, Oliver Van Hecke, Femke Wauters, Eva De Leeuw, Janique Lobbestael, Sara Willems

**Affiliations:** a Department of Emergency Medicine, Ghent University Hospital, Gent, Belgium; b Department of Public Health and Primary Care, Ghent University, Ghent, Belgium; c Directieteam ICHO (Interuniversitair Centrum voor HuisartsOpleiding), Leuven, Belgium

**Keywords:** Unplanned care, out-of-hours primary care, emergency department, patient trajectory, healthcare organisation

## Abstract

**Background:**

Demand for unplanned care in Belgium is high, placing pressure on both out-of-hours primary care facilities (OOHPCF) and emergency departments (ED). Understanding patient trajectories may help optimise care pathways and resource planning for planned and unplanned care.

**Objective:**

To describe patient presentations and trajectories at OOHPCF and EDs in a region of Flanders, Belgium.

**Methods:**

Data were collected in six EDs and three OOHPCF in the greater {redacted} region. All consultations were recorded during three randomly selected days in EDs and one full week in OOHPCF in 2024.

**Results:**

A total of 1,469 ED and 857 OOHPCF consultations were analysed. In EDs, trauma was the most common presentation (32%), followed by ear, nose and throat (ENT) and dermatology conditions (22%). In OOHPCF, trauma and orthopaedic problems accounted for 20% of consultations, followed by ENT (17%) and dermatology (14%). Most ED patients (71%) were discharged home. Self-referral accounted for 65% of ED visits; these patients were generally younger and presented with lower-acuity conditions. One in five patients had consulted another healthcare provider within the previous week. Pain and worry were the main drivers for attendance. Attending clinicians in both settings considered up to one-third of consultations manageable in planned care.

**Conclusion:**

Rather than restricting access or apportioning blame to patients, our findings indicate the need to reassess measures throughout planned and unplanned care pathways for timely access to the most appropriate care setting while maintaining patient safety and equitable access to services.

## KEY MESSAGES

- One third of unplanned care consultations were perceived as manageable in planned care.
- Key drivers for unplanned care were pain and patient worry about their symptoms.
- Our findings signal the need to re-evaluate strategies for timely access to appropriate care while maintaining patient safety and equitable access to services.

## Introduction

Despite advances in healthcare, the demand for unplanned care keeps increasing, creating an unprecedented challenge for both out-of-hours primary care facilities (OOHPCF) and emergency departments [ED] across most high-income countries. Several factors contribute to this rising demand: an ageing population, the expansion of ambulatory care for complex acute and chronic conditions, the reduced availability of scheduled (planned) care in both primary care and hospital settings, and evolving patient preferences and expectations for expedient care [1–6]. The problems are mutual and linked.

In Flanders, Belgium, ED consultations rose by 23% between 2010 and 2019, reaching 246 contacts per 1,000 citizens annually. Rates in Brussels and Wallonia are even higher [7]. Similar increases were observed in OOHPCF [8]. In response to this rising demand and escalating societal costs, decision-makers in Belgium identified a range of service models, mostly to divert patients to alternative care pathways. One such example -- similar to initiatives in neighbouring countries -- is co-locating general practitioners [GPs] within so-called community care centres in or alongside the ED as key providers of out-of-hours primary care [2]. The true impact of introducing such initiatives on ED use, patient satisfaction, and cost-effectiveness remains unproven. Hence, ED overcrowding is at least as much the result of throughput (increased patient complexity, stringent quality indicators), output (boarding delays, socioeconomic barriers) and bottlenecks due to increased input (patient attendance) [3–5,9–11]. This framework is widely known as the Input--Throughput--Output (ITO) model of emergency department crowding and patient flow.

The current Belgian healthcare system lacks a formal gatekeeping mechanism for ED access. Various studies have estimated that up to half of all ED visits in Belgium may be grouped as ‘non-urgent ED attendance’, or ‘potentially avoidable ED visit’ [1,2,12]. However, as in international literature, these studies struggle to determine unequivocally what constitutes such a ‘potentially avoidable ED visit’ and is dependent on the context and professional background. Discussions (in Belgium) are often fuelled by assumptions and misconceptions – (e.g. deliberate misuse, convenience, specific subgroups being overrepresented) -- driven by international data (at best of low certainty) and/or opinion.

While increased input is not the sole driver of ED or OOHPCF crowding, improving the actual patient trajectory at regional level could have a positive impact on patient and healthcare professional satisfaction, the quality of care provided, and cost-effectiveness. This demands an important restructuring of the system of unplanned care. Understanding the patient trajectory at the regional level will provide key insights to guide Belgian policymakers into how care pathways can be optimised to better plan for unplanned and planned care.

## Methods

As part of quality improvement efforts in the wider {redacted} region, all four EDs in the city centre {redacted} together with two regional EDs in more rural areas {redacted}, prospectively collected additional data alongside their routine clinical workload. Data were gathered for all consultations during three randomly selected 24-hour periods (one Monday, one Thursday, and one Saturday) between March and June 2024. The additional data captured included reasons for presentation, language and ease of communication, referral pathways, and recent healthcare contacts. Furthermore, the treating physician independently assessed whether each consultation could have been managed in OOHPC or within a planned care setting. Patients were considered to be self-referred if they were not admitted after emergency medical services [EMS] intervention or not referred by a healthcare provider after physical or telephonic contact or written advice. Data were fully anonymised and entered into a central database. Patients were informed of this by means of a poster displayed in the waiting room of each ED. The study was approved by the ethics committee of {redacted}. Given the nature of this study, a waiver of consent was granted.

In parallel, GPs from the two OOHPCFs in {redacted} {redacted} and the one in {redacted} also recorded these additional data points for each contact. All three OOHPCFs are situated on a hospital campus, close to the ED, and open only at weekends and weeknights (6 pm-8am). One of the two OOHPCFs in {redacted} (UH) has limited opening hours, closing at 11 pm. For the OOHPCFs, data were collected from one randomly chosen 7-day week and entered into an anonymised database. Patients were also informed about the study by a poster in the waiting room.

The primary investigators conducted descriptive analysis of the data. Missing or incorrect/illegible data were excluded from further analysis (see supplement 1). Data are reported as medians (with interquartile range [IQR]), means (with standard deviation [SD]) or sample percentages. Where informative, 95% confidence intervals [CI] for mean (T-test), median (Mann-Whitney U) or proportional differences [95% CI prop. diff.] (Miettinen-Nurminen method, *via* Statsdirect 3.3.6) were calculated.

## Results

Our total sample consisted of 1469 ED consultations and 857 OOHPCF consultations.

The median time of consultation was 15h (IQR 11;18) for ED and 14h (IQR 10;19) for OOHPCF consultations. For ED, the mean age of patients consulting was 41.4 y (± 25.6); for OOHPCF, it was 31.6 y (± 25.1). The population in the OOHPCF sample was significantly younger (95% CI diff. means: −11.9 y to −7.5 y) than that in the ED. Ninety-two per cent of the patients in the ED sample had a dedicated GP; in the OOHPCF sample, this was 96.9%.

The most frequent primary complaints in the ED sample were trauma (32%), gastroenterology (11.5%) and other problems, including Ear-Nose-Throat [ENT] & dermatology (22.3%). For the OOHPCF, this was similar: trauma & orthopaedic problems accounting for 19.7%, ENT for 16.5%, and dermatology for 14.1% (see Table 1). The distribution of primary complaints was relatively similar across the participating EDs, apart from the University Hospital ED with relatively less trauma and more internal pathology, as well as significantly more psychiatric problems (4% more; 95% CI proport. diff. 1.5–7.5).

**Table 1.** Primary complaints registered for consultations in emergency department and out-of-hours primary care facilities.

Primary complaints	ED % (*N* = 1451)	OOHPCF % (*N* = 843)
Trauma	32	10
Orthopaedic problems	N/A*	9.7
Ear-nose-throat (ENT)	N/A*	16.5
Dermatology	N/A*	14.1
Gastro-enterology	11.5	12.2
Infectious	7.6	N/A*
Pneumology/Respiratory	5.9	10.7
Cardiology	5.7	3
Neurology	5.2	3.5
Urology – Gynaecology	6	6.7
Psychiatric	3.8	1.4
Other	22.3	10.9

ED: emergency department; OOHPC: out-of-hours primary care facilities; *There is overlap and certain complaints are not applicable [N/A] for one of the settings.

In the ED sample, patients were triaged by severity using an adjusted Manchester Triage System. A total of 9.3% were considered high acuity (red-orange), 40.7% moderate (yellow) and 50% low (green-blue). Triage level blue was observed in only 4.7% (very low acuity; see Figure 1). The University hospital had significantly more patients triaged as high acuity (8.6% more; 95%CI proport. diff. 4.5-13.4%). No triage tool was used in the OOHPCF.

**Figure 1.**
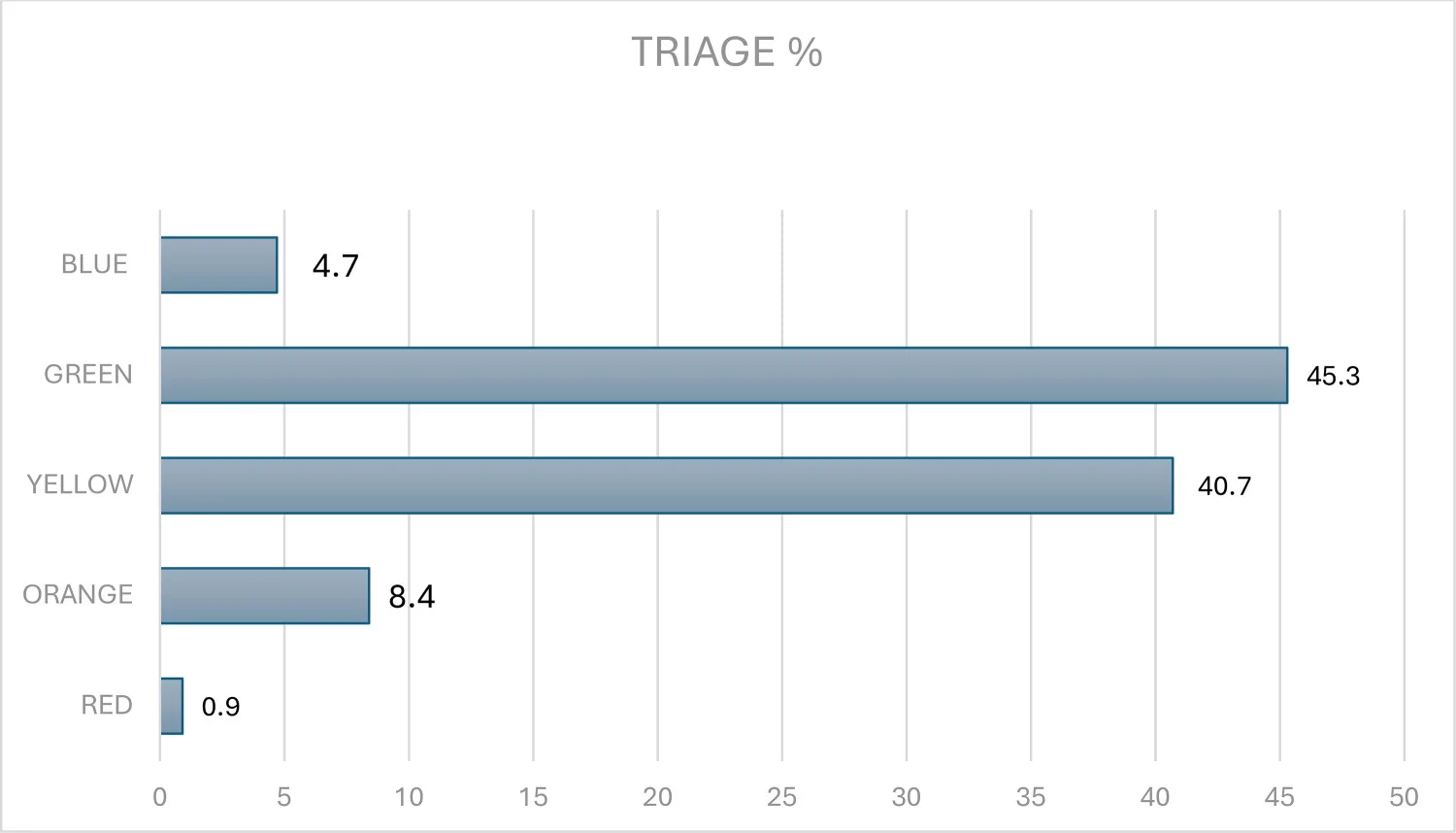
Triage colour of all ED patients as per Manchester Triage System (% of total). Blue (non-urgent), green (standard), yellow (urgent), orange (very urgent), red (immediate).

Overall, 65.1% of ED consultations were self-referred. Self-referred patients were a significantly distinct population compared with those referred (Table 2). They were younger, had more low-acuity problems (many of which had minor trauma as primary complaint) and were less likely to be admitted to the hospital. Their native language was less likely to be Dutch. The reasons for the encounter among self-referred patients at the ED were mostly related to pain (60.3%) or worry (41.4%). In addition, many patients reported rapidness (27.6%) or accessibility (10.8%) as the main drivers of their ED visits. Not knowing the other care alternatives (4.5%) or financial motives (0.6%) were rare. Self-referral after failure to secure a consultation in a planned care setting was reported in 5.7% of ED and 2.5% of OOHPCF patients.

**Table 2.** Difference in key factors between those presenting to emergency departments after referral (excl. those after emergency medical services intervention) and those considered to be self-referred.

ED patients (*N* = 1459)	Self-Referral [SR]	Referred patients	95% CI For Diff. means/proportions
Time of presentation (hour, median-IQR)	15 (11–19)	14 (11–18)	/
Age (y, mean-SD)	37.3 (23.7)	49 (27.7)	SR 8.6 to 14.7 y younger
Primary complaint: Trauma (%)	39.2	13.6	SR 20.5 to 29.7% more
Low acuity Triage (blue-green, %)	56.9	33.4	SR 17.8 to 28.9% more
Native language: Dutch (%)	72.6	86.9	SR 9.7 to 18.5% less
Discharged with/without follow-up (%)	44.7 / 34.8	38.2 / 15.2	SR 14.9 to 24.1% more without follow-up
Hospital admission (incl. ICU, %)	20.5	46.9	SR 20.9 to 31.8% less
‘This patient should have been managed in planned care setting’ (%)**	33.1	24.8	SR 3.1 to 13.3% more
‘This patient should have been managed in OOHPCF’ (%)**	20	8	SR 8.1 to 15.5% more
‘This patient should be evaluated in the ED regardless/ (%)**	53.5	77.2	SR 18.4 to 28.7 less
‘If possible, I would have referred this patient to OOHPCF (%)**	45.2	19.9	SR 20.2 to 30.2% more

ED: emergency department; OOHPC: out-of-hours primary care facilities; ** The last four discriminators are subjective assessments by the treating physician.

Of those ED patients who self-referred, 21.2% had a recent consultation in the last week. Among those who were referred, this was 38.6%. This was significantly higher than in the OOHPCF where only 16.7% had such contact in the last week.

Dutch was the mother tongue of 91.2% of patients presenting at the ED and of 79.3% of the patients presenting at OOHPCF consultations (95% CI prop. diff. 8.8 to 15.1% lower in OOHPCF). Although there was often an accompanying person or relative to facilitate communication, the treating physician still considered the communication to be moderately to severely difficult in 15.3% of ED and 5.7% of OOHPCF consultations, respectively (95% CI prop. diff. 7.1 to 12% higher in ED). The more difficult the communication was perceived, the less likely the consultation was deemed appropriate, both for the ED sample (95% CI prop. diff. 0.4 to 15% less) and the OOHPCF sample (95% CI prop. diff. 6.3 to 32.1% less).

Regarding the outcomes of the consultation at the ED, 70.5% of patients were discharged, of whom 41.8% required no further follow-up, 22.6% were admitted to a ward, 3.7% to critical care, and 3.2% to the operating room (see Table 3). The discharge rate was highest among patients with trauma as their primary complaint (89.5%) and lowest for cardiology or pneumology problems (44.6%). Two of the patients died in the ED.

**Table 3.** Difference (in %) for key factors -outcome and perception about trajectory- between those presenting to emergency department and out-of-hours primary care facilities.

	ED (*N* = 1451)	OOHPCF (*N* = 844)
Outcome:		
Ambulatory without follow-up %	28.7	52
Ambulatory with follow-up %	41.8	35.3
ED-Radiology %	n/a	8.6
Hospital Admission %	29.5	unknown
Perception of attending physician about optimal patient trajectory^±^		
ED %	54.5	1.8
OOHPCF %	15.9	66
‘PLANNED CARE’ %	29.5	29.9
No professional care needed %	Not reported	2.4

ED: emergency department; OOHPC: out-of-hours primary care facilities.

^±^The last four discriminators are subjective assessments by the treating physician.

In 15.9% of ED consultations, the treating physician thought that the patient could have been treated equally well in the OOHPCF and another 29.5% in a planned care setting. Eventually, in 54.5% of consultations, the treating physician considered the ED choice fully appropriate. When the question was reframed – if allowed and available -- the same physician would have diverted the patient from the ED to an OOHPCF in 36.1% of their patients.

Reasons for consulting an OOHPCF were pain (46.3%), worry (52.5%), rapidness (22%), and accessibility (13.2%). Administrative requests (e.g. a sickness note) accounted for 7.2% of all consultations. After consultation, 6.6% were referred to the ED and 2% to radiology. Approximately half (52%) of the patients were discharged without further follow-up in planned care. The attending physician considered the OOHPCF consultation appropriate in 66%; 29.9% could have been managed in planned care; 1.8% should be seen in the ED; and 2.4% did not require any clinical intervention. Patients who had a medical contact in the last week (16.7%) or tried to get an appointment but failed (2.5%) were more likely to have a consultation considered inappropriate (28.1% vs 14.5%; 95% CI prop. diff. 7.8 to 19.7% higher).

Table 3 presents the outcomes of the consultations and the appropriateness assessment in both samples. While most indicators differed between groups, the number of patients that presumably would have been better served in a planned care setting was very similar across both groups.

## Discussion

In our study, a third of consultations in ED and OOHPCF were perceived as neither time-sensitive nor pathologically urgent and were likely to be managed in a planned care setting (e.g. scheduled primary or secondary/tertiary care). Pain and worry were the predominant drivers for consulting. Although there is a temptation to set-up systems restricting access or apportioning blame to patients, our findings signal the need to carefully rethink and evaluate strategies for timely access to the most appropriate care setting while maintaining patient safety and equitable access to services.

The number of (perceived) ‘potentially avoidable ED visits’ in our study concur with existing literature (20-50%). However, definitions of avoidable ED visits differ across studies, relying on factors such as triage urgency, expected resource use, patient outcomes, and subjective assessments [4,9,12–19]. Defining a ‘potentially avoidable ED visit’ remains inherently complex and context-dependent, often influenced by the professional background and clinical setting of the healthcare providers involved. Moreover, the proportion of patients redirected away from the ED following triage is often far less than theoretical estimates [4,20].

In identifying this population, most studies focus on self-referred patients and the motivations behind their decision to seek care. Contrary to what is often stated in public discourse, media, and policy circles, low-certainty evidence suggests that these decisions are not primarily driven by convenience or limited access to other providers. Instead, patients often cite acute symptoms such as pain, concern about potentially serious conditions, and a strong belief that the ED or OOHPCF is the most appropriate venue to address their concerns [15,21,22]. A patient’s decision to attend unplanned care may still have been entirely reasonable. This pattern was also seen in our study, where pain and worry were the most frequently reported reasons for consulting. Interestingly, although many characteristics between the two populations were very similar, the OOHPCF sample was younger and had less prior physical healthcare contact in the preceding week (1 in 6 patients).

Comparing data across regions or countries regarding patient trajectories and resource utilisation in EDs and OOHPCF—whether for unplanned medical issues or not—is inherently difficult due to the significant influence of each region’s healthcare organisation [3,10,23–25]. For instance, in a Swedish ED sample, only 34% of patients were self-referred, and 22% were triaged as high acuity. Our ED sample showed a markedly different pattern, with about two-thirds of patients self-referring and less than 10% receiving a high-acuity triage score. Such differences are often cited as arguments in favour of stricter gate control for EDs and OOHPCFs, by means of mandatory triage—whether by telephone or in person. The test performance of existing triage scores and/or predictive tools, and the presumed benefits of diverting low-acuity patients away from EDs and other ‘unplanned care’ settings remain unproven. Moreover, setting up a gate triage system is resource-intensive and does not necessarily correlate with reduced subsequent healthcare utilisation or other important outcomes [1,3,4,9,12,17,26].

In our study, treating ED physicians would, if possible, divert more than a third of presenting patients. Importantly, none of the commonly used indicators—such as triage colour (low-acuity), time of presentation, self-referral status, reason for attendance, primary complaint, or an ‘ambulatory’ outcome—demonstrated sufficient test performance to reliably identify this subpopulation. Therefore, as suggested in available literature, developing a triage system with both high specificity and sensitivity is not straightforward nor without risk. Policy makers should be aware of this. This does not, however, take away that the diversion of these patients from either ED or OOHPCF will not necessarily result in a proportional reduction in staffing costs or resource allocation, which are often still needed in equal measure for the remaining moderate- to high-acuity patients [3,4,9,14,17,26,27].

Does this mean there are no meaningful gains to be had by guiding patients towards more appropriate care trajectories—ensuring ‘the right patient receives the right care at the right time’? Based on our findings, optimising care pathways appears feasible in some presentations. Evidence suggests this could increase patient satisfaction and caregiver job satisfaction (and reduce burnout) and might provide a safety net when planned care options fall short [3,4,9,17,27,28]. If integrated into a larger plan that also tackles processes and output in the ED/OOHPCF setting, this might help curtail healthcare costs by e.g. avoiding duplicate consultations and unnecessary or repeat testing [3,4,9,14,17].

We acknowledge our study has limitations. We used a convenience sample from one region of Flanders within a limited time-period to avoid overburdening clinical staff. Although we are confident that this provides a reasonable reflection of the broader {redacted} region, we cannot be sure that these data are representative of Flanders, let alone Belgium. Repeating the study at a different time (e.g. the winter season) or in a different region may yield different results. Nonetheless, other Belgian demographic data on ED attendance appear relatively consistent with our findings [7,12,17,22]. Our study relied on the treating physician’s subjective assessment of each consultation, without insight into the reasoning behind these judgments. Another limitation is the reliance on information available after assessment or after the final diagnosis is known. A patient presenting with chest pain, severe headache, or shortness of breath may ultimately have a benign condition, but their decision to attend the ED may still have been entirely reasonable. Moreover, further qualitative research is needed to explore patients’ motivations or barriers (e.g. psychosocial, geographical) that may have influenced their care-seeking behaviour. Yet, although clinician perceptions of a ‘potentially avoidable ED attendance’ may not accurately reflect the validity of patients’ care-seeking decisions, they remain important because they influence clinical interactions, staff wellbeing, service design, and healthcare policy. Furthermore, such perceptions may provide valuable insight into broader healthcare system deficiencies and the disconnect between patient and clinician understandings of urgent care need.

Finally, data collection was conducted as part of routine practice, which might have affected quality. Although there was limited missing data (see Appendix 1), we did not implement specific quality assurance measures for the registered data. Each registering site had a local coordinator who coached the team in completing all data during the audit days.

## Conclusion

Our study provides insight into patient trajectory for ‘unplanned care’ in one region of Flanders with a population of just under 500,000. Pain and worry about symptoms were the main drivers for attendance. Although a substantial proportion of unplanned care visits were perceived as manageable in planned care, we should resist apportioning blame to patients and/or restricting access but rather holistically reassess the interplay between components of unplanned care (input, throughput, output) to optimise care pathways in both planned and unplanned care.

## Supplementary Material

Supplemental Material
