# Supplementary material for: Describing the patient trajectory for unplanned care in a region of Flanders, Belgium: A prospective observational study
Source: Eur J Gen Pract. 2026 Oct 26;32(1):2745925. doi: 10.1080/13814788.2026.2745925 (PMC13715331; doi:10.1080/13814788.2026.2745925)
Supplement: Supplementary file 1 [file IGEN_A_2745925_SM4764.docx]

**SUPPLEMENT 1**

| MISSING DATA | ED SAMPLE (N=1469) | OOHPCF SAMPLE (N=857) |
| --- | --- | --- |
| HOUR | 4 | 60 |
| AGE | 8 | 46 |
| DAY | 0 | 15 |
| MAIN PROBLEM | 18 | 14 |
| TRIAGE COLOUR | 47 | N/A |
| REFERRAL? | 10 | 37 |
| REASON PRESENTATION | 3 | 15 |
| RECENT CONSULT | 42 | 17 |
| GMD GP | 15 | 22 |
| NATIVE LANGUAGE | 31 | 26 |
| EASE of COMMUNICATION  (patient + accompanist) | 76 | 36 |
| OUTCOME | 18 | 13 |
| APPROPRIATENESS EVALUATION | 17 | 10 |

Supplement table A: Missing or incorrect/illegible data for each of the datapoints, per group (ED or OOHPCF sample)
